# Mechanically robust amino acid crystals as fiber-optic transducers and wide bandpass filters for optical communication in the near-infrared

**DOI:** 10.1038/s41467-021-21324-y

**Published:** 2021-02-26

**Authors:** Durga Prasad Karothu, Ghada Dushaq, Ejaz Ahmed, Luca Catalano, Srujana Polavaram, Rodrigo Ferreira, Liang Li, Sharmarke Mohamed, Mahmoud Rasras, Panče Naumov

**Affiliations:** 1 Smart Materials Lab, New York University Abu Dhabi, Abu Dhabi, UAE; 2 Division of Engineering, New York University Abu Dhabi, Abu Dhabi, UAE; 3 Department of Chemistry, Khalifa University of Science and Technology, Abu Dhabi, UAE; 4 Radcliffe Institute for Advanced Study, Harvard University, Cambridge, MA USA

**Keywords:** Mechanical properties, Optical materials, Fibre optics and optical communications

## Abstract

Organic crystals are emerging as mechanically compliant, light-weight and chemically versatile alternatives to the commonly used silica and polymer waveguides. However, the previously reported organic crystals were shown to be able to transmit visible light, whereas actual implementation in telecommunication devices requires transparency in the near-infrared spectral range. Here we demonstrate that single crystals of the amino acid L-threonine could be used as optical waveguides and filters with high mechanical and thermal robustness for transduction of signals in the telecommunications range. On their (00$\bar 1$) face, crystals of this material have an extraordinarily high Young’s modulus (40.95 ± 1.03 GPa) and hardness (1.98 ± 0.11 GPa) for an organic crystal. First-principles density functional theory calculations, used in conjunction with analysis of the energy frameworks to correlate the structure with the anisotropy in the Young’s modulus, showed that the high stiffness arises as a consequence of the strong charge-assisted hydrogen bonds between the zwitterions. The crystals have low optical loss in the O, E, S and C bands of the spectrum (1250−1600 nm), while they effectively block infrared light below 1200 nm. This property favors these and possibly other related organic crystals as all-organic fiber-optic waveguides and filters for transduction of information.

## Abstract (web summary)

Fiber-optics based on organic crystals could have potential for unique telecommunications applications but typically transmit visible wavelengths. Here the authors present mechanically robust organic crystals with favourable optical properties across the main telecommunication bands in the near-infrared.

## Introduction

With the increased security threats inherent to the wired (electron-based) transfer of information and the explosive expansion of the internet traffic in recent decades, there is a rising need for fiber-optic materials that are safe, robust, light and durable. The currently used commercial fiber-optics are based on cladded silica of high purity, and are produced in massive amounts for inter-continental optical cables through which nearly all voice, video, and data communications are transferred instantaneously around the globe. One of the shortcomings of the silica fibers is their performance with highly intense light, which poses challenges with transfer of high-definition data required by the internet and cellular service providers, particularly in view of the ongoing transition to the fifth generation (5G) wireless technology and high-performing phone networks. Organic polymers, which have also been used as waveguides, are light in weight and can be easily processed, however, their opaqueness results in significant signal attenuation. Specific applications, particularly on a small scale, such as in optoelectronic components in micro/nanocircuits, require alternative materials where the performance would compensate the cost due to their small size. As some of the main prerequisites that stand before new materials for this purpose, their optical and other physical assets, and most importantly mechanical properties, should be chemically accessible and tunable.

Single crystals of small organic compounds have been recently considered viable candidates for such applications and have been actively researched in the past several years^1–5^. The studies have focused on the demonstration of passive (transmission of unaltered input light) and active (transmission of fluorescence) transduction of visible light, which has been already demonstrated for a number of organic crystalline materials^2–28^. The mechanical compliance observed with some of these elastic and plastic materials is unprecedented and impressive^7–12^, although intentional (for applications that require bending of the light beam) or unintentional (due to accident or damage) deformation of their crystals is usually accompanied with accumulation of defects^20^. Although their shape can be recovered, the defects result in proneness to wear and fatigue, and decrease drastically the optical transmittance after multiple deformations. The most recent efforts in this research endeavor have been directed towards sequencing, welding, combining, and scaling down of prospective organic crystalline waveguides, as well as development of methods for their micromanipulation and incorporation in micro-optical devices^26–29^ and other electronics^30,31^. Although these developments have undoubtedly demonstrated the potential of organic crystals as organic waveguides, without exception they have focused on the visible part of the spectrum (usually, 500−600 nm), which does not immediately appeal for transduction of communication signals. Indeed, the current fiber-optic communication networks require the transmission of light with low optical loss in the 1260−1625 nm region, which for the purpose of quick reference to the signal energy is divided into five wavelength bands referred to as the O, E, S, C, and L bands. To our knowledge, organic crystals of small molecules have not been explored as optical transducers in this region.

**Fig. 1:**
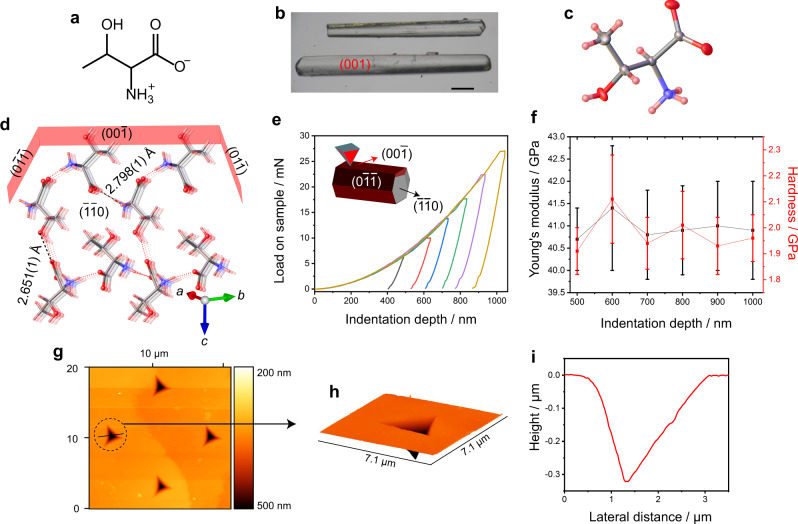
Structure and mechanical properties of L-threonine crystals. **a** Chemical structure showing the zwitterionic form of threonine (the stereochemistry is not shown). **b** Optical microscopic image of typical crystals under study. The length of the scale bar is 500 μm. **c** ORTEP diagram of L-threonine shown at 50% ellipsoid probability level. **d** Molecular packing diagram showing head‐to‐tail packing in the structure of L-threonine and hydrogen bonds as broken lines. **e** Load–depth curves recorded from an L-threonine crystal on its (00$\bar 1$) face at different penetration depths. **f** Young’s modulus (*E*) and hardness (*H*) based on the curves similar to those shown in (**e**). The error bars show the standard deviations that were calculated from 25 indents at each indentation depth. **g** An example of AFM topography images of the indent impressions. **h** Topography of a typical indent showing absence of material pileup. **i** Height profile of the indent shown in (**h**).

Here we report that single crystals of the amino acid L-threonine (2-amino-3-hydroxybutanoic acid; Fig. 1a), which are endowed with exceptional mechanical robustness, can be used as a prototypic organic waveguide and a filter in the near-infrared region of the spectrum. In addition to these crystals being advantageous for being lighter (*ρ* = 1.3 ± 0.1 g cm^−3^) than silica (*ρ* = 2.65 g cm^−3^), unlike silica they also come with the possibility for chemical modifications that would alter the optical and other physical properties of the ensuing organic waveguides. This novel result of using stiff and hard organic crystals as optical waveguides in the infrared range opens up prospects for application of small-molecule organic crystals with high stiffness and hardness as durable signal-transducing media.

## Results

For the purpose of this study, single crystals of L-threonine were grown by slow evaporation from an aqueous solution as elongated prisms of up to about 10 mm in length and a cross-section of about 5 mm^2^. The single crystals were colorless, clear and devoid of visible defects (Fig. 1b, Supplementary Fig. 1). The crystal structure, which for the purpose of this study was re-determined at room temperature by using single crystal X-ray diffraction (Supplementary Table 1), showed that they are in the chiral orthorhombic space group *P*2_1_2_1_2_1_ with one molecule in the asymmetric unit (Fig. 1c). The zwitterions are bonded with each other via two strong hydrogen bonds (*d*(O···O) = 2.651(1) Å, *d*(N···O) = 2.798(1) Å; Fig. 1d). The nanomechanical properties of the crystals were determined by nanoindentation (Fig. 1e−i; Supplementary Figs. 2−4). The stiffness (the extent to which an object resists elastic deformation in response to an applied force) and the hardness (the surface resistance of the material to pressure or scratching with a sharp object) were determined by nanoindentation. The accessible crystal face(s) where they could be indented were matched with their Bravais–Friedel–Donnay–Harker (BFDH) morphology that was reconstructed from their crystal structure. The load–displacement curves obtained at varying penetration depths on the (001)/(00$\bar 1$) face and the residual impressions imaged by atomic force microscopy (AFM) are shown in Fig. 1e−i. The profiles are smooth and do not suggest slippage of molecular layers. The elastic modulus on (00$\bar 1$) was found to be *E* = 40.95 ± 1.03 GPa for indentation depths between 500 and 1000 nm and a total of 150 indents. On their ($\bar 1\bar 1$0) and ($0\bar 1\bar 1$) faces, the measured values of the moduli are *E* = 25.33 ± 1.37 GPa (150 indents) and 32.57 ± 0.67 GPa (150 indents), respectively (Supplementary Figs. 3 and 4). The modulus on the (00$\bar 1$) face is particularly high; it is significantly higher than typical values for organic crystals, which have values 1─25 GPa with about 8% < 1 GPa and about 8% >25 GPa, and it is comparable to other amino acids and sugars having strong hydrogen bonding networks. Prominent organic materials with very high modulus include β-succinic acid (*E* ≈ 126 GPa^32,33^; note, however, that this value was obtained by ultrasonic resonance measurements and is not directly comparable to the values reported here), a cyclic dipeptide (*E* = 20.5 ± 0.66 GPa)^34^, sucrose (*E* = 33 and 38 GPa)^35^, a cocrystal of caffeine and a benzoic acid derivative (*E* = 76.86 and 64.39 GPa)^36^, and α-glycine (*E* = 26–44 GPa, depending on the crystal face)^37^. At *H* = 1.98 ± 0.11 GPa for the (00$\bar 1$) face, the hardness of L-threonine is also exceedingly high for an organic compound, and is higher than those of many organic crystals (typical range of hardness for organic crystals is ~0.1–1.5 GPa)^38–42^.

**Fig. 2:**
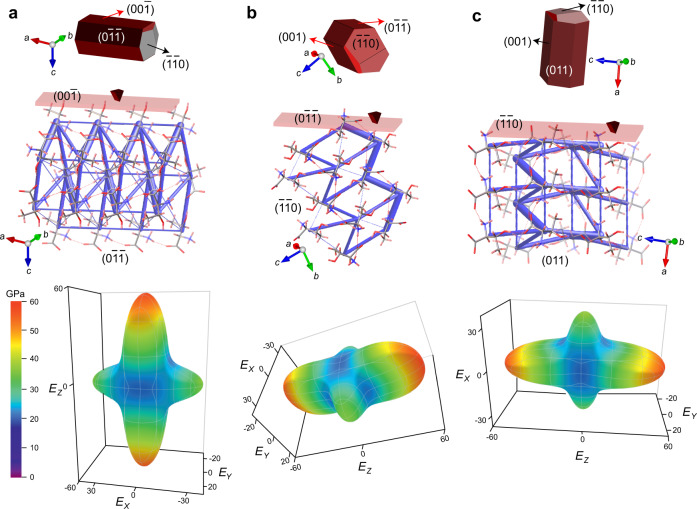
Face indices, energy frameworks, and computed 3D surfaces of the Young’s modulus for L-threonine crystal. The properties are shown as viewed along faces: **a** (00$\bar 1$), **b** (0$\bar 1\bar 1$), and **c** ($\bar 1\bar 1$0). Only the interaction energy component of the energy frameworks is shown as blue sticks. The thickness of each tube is proportional to the strength of the respective interaction. The scaling of the tubes is the same in all pictures. To improve the clarity of the pictures, the frameworks and crystal packing were reconstructed and merged, and interaction cutoff of 5 kJ mol^−1^ was applied. The color-coded scale applies to all three plots of the Young’s modulus.

The exceptional mechanical properties of the L-threonine crystals observed were rationalized through structure–mechanical property relationships that were established by analysis of the energy frameworks (Fig. 2)^43^. The pairwise interaction energies in the crystal structure of L-threonine are represented as cylinders connecting the zwitterions, and the cylinder radii are directly proportional to the respective relative strengths of the intermolecular interactions (the individual contributions to the interaction energy are provided as Supplementary Fig. 5). The interaction topologies of the zwitterions in L-threonine indicate the occurrence of strong interactions and in the plots of the energy frameworks appear as larger tubes. As can be inferred from the plots of the total interaction energy in Fig. 2, the closely packed molecular arrangement with strong hydrogen bonding interactions and the relatively uniformly distributed energy of the intermolecular interactions are the main reasons behind the extraordinarily high stiffness and hardness of this material. Specifically, as shown in Fig. 2a, the exceptional stiffness on the (00$\bar 1$) face corresponds with the energy contributors that are aligned close to the direction perpendicular to that face. These further reflect the collective strength of the hydrogen bonds in the structure, and confirm that the exceptional mechanical properties are due to the strong intermolecular charge-assisted hydrogen bonds. This conclusion is in line with other reports of amino acids or peptides of exceptional stiffness^37,42^.

First principles density functional theory (DFT) methods are frequently applied for computing the mechanical properties of inorganic minerals^44^. However, within the emerging field of crystal adaptronics^45,46^, such computations are relatively rare for molecular solids and most reports of mechanical properties of organic materials are derived from experimental nanoindentation measurements on accessible crystal faces. Nevertheless, we envisage that as more examples of mechanically compliant organic crystalline materials are reported, computed mechanical properties will play an important role in rationalizing the properties of such materials and guiding the discovery of novel materials with useful solid-state properties. Here, we apply the stress-strain method^47^ using optimized high efficiency strain-matrix sets (OHESS) coupled with state-of-the-art DFT methods to compute the second-order elastic constants of L-threonine. The computed Young’s modulus has a range of *E* = 20.53–57.66 GPa with an average value of 33.11 GPa. In view of the neglect of thermal effects in the computations, the calculated average *E* value is within an acceptable error threshold of the experimentally determined value of 40.95 ± 1.03 GPa for the (00$\bar 1$) face. We also note that the experimental *E* values for all faces are within the range predicted by the calculations. The calculations reveal a strong anisotropy in the Young’s modulus surface as a function of the crystallographic axes (Fig. 2), with the maximum value of *E* observed along the crystallographic *c* axis. Indeed, the *C*_33_ elastic constant has a value of 74.27 GPa, the highest of all 9 computed elastic constants for this orthorhombic crystal (Supplementary Table 2). In accord with the conclusions based on the interaction energies above, the anisotropy of the Young’s modulus arises as a consequence of the directional charge-assisted hydrogen bonds of L-threonine which propagate parallel to the (001) plane of the crystal (Fig. 2). The computed molecular electrostatic potential (MEP) of L-threonine (Supplementary Fig. 6) shows that the relatively small surface area of the zwitterion produces highly localized potential energy values ranging from +241 kJ mol^−1^ to −276 kJ mol^−1^, leading to strong complementary charge-assisted hydrogen bonding interactions in the crystal. We conclude that the exceptional hardness and Young’s modulus of this organic material arise as a consequence of the favorable ionization of L-threonine in the crystal, leading to strong charge-assisted hydrogen bonds propagating along the *c* axis.

**Fig. 3:**
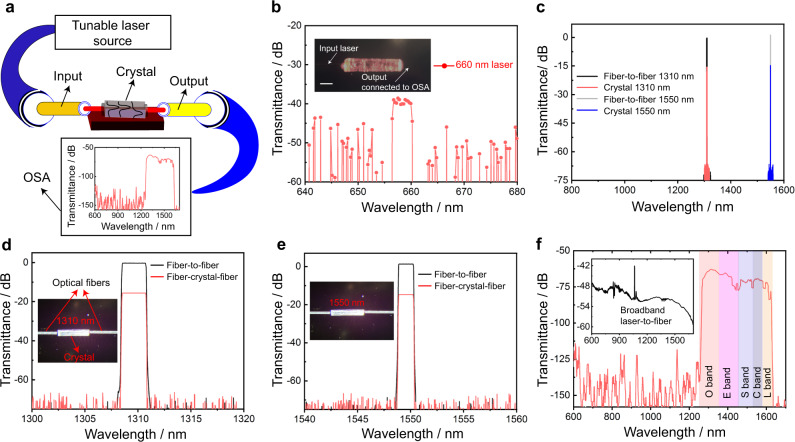
Optical waveguiding properties of L-threonine crystals. **a** Schematic representation of waveguide analysis of a crystal by using optical spectrum analyzer (OSA). **b** Optical losses in the visible region. The length of the scale bar in the inset is 700 μm. **c** Optical spectra of the output at 1310 nm and 1550 nm laser excitation sources. **d**, **e** Optical spectrum in datacom regime obtained by using 1310 nm (**d**) and 1550 nm (**e**) lasers with a fiber-to-fiber coupling as a reference. **f** Broadband response of the crystal in the 600–1700 nm range showing light transduction in the 1250–1600 nm window, inset showing broadband laser to fiber reference. The bands are labeled with their common acronyms (O—“original band”, E—“extended band”, S—“short wavelength band”, C—“conventional band”, L—“long wavelength band”).

The optical waveguiding properties of the L-threonine crystals were tested by coupling a single crystal with a suitable input from different light sources (Fig. 3a). In order to reduce light scattering and insertion losses between the fiber and the crystal, clear crystals of good quality with sharp edges were used. Using a micromanipulator, one end of the prismatic crystal was connected to a single-mode FC/PC fiber-optic patch cable which was further connected to a 4-channel fiber-coupled laser source (MCLS1 Thorlabs) set at a maximum laser drive current (100 mA) at 660 and 1064 nm. The light coming out from the other end of the crystal was collected with another single-mode FC/PC fiber optic patch cable that was connected to an optical spectral analyzer (Yokogawa AQ6370D) operating in the range 600–1700 nm. The average dimensions of the crystals used in these experiments were 2.9 ± 0.2 mm in length, 0.6 ± 0.1 mm in width, and 0.3 ± 0.1 mm in height. When a crystal is excited at 660 nm or 1064 nm at one end, it transmits light to the opposite end (the spectrum at 660 nm is shown in Fig. 3b). The optical losses due to partial reabsorption of light by the crystal measured at laser excitation of 660 nm and 1064 nm are 40 dB and 22 dB, respectively (considering the length of the measured crystals, the losses are 13.7 ± 1.2 dB mm^–1^ and 7.5 ± 0.6 dB mm^–1^ at laser excitation of 660 nm and 1064 nm, respectively). The drastic decrease in optical loss at 1064 nm relative to that at 660 nm indicated that the crystal was more transducive at high wavelengths, and therefore its waveguide capabilities in the optical communication range of frequencies were examined further. Supplementary Fig. 8 shows that the optical loss decreases with increasing excitation wavelength.

A tunable laser source (Keysight 8164B Lightwave Measurement System) was used as input with wavelengths in the near-IR region, specifically the O band (1260–1360 nm) and the C band (1530–1565 nm), which are commonly used in standard transmission measurements through glass-based fibers (Fig. 3c). Figures 3d and 3e show the optical spectra of the crystal output for 1310 nm and 1550 nm laser excitation along with the fiber-to-fiber calibration as a reference. Remarkably, the crystal exhibits very low losses of about 15.58 dB and 14.69 dB at excitation of 1310 nm and 1550 nm, respectively (considering the length of the measured crystals, the losses are 5.3 ± 0.4 dB mm^–1^ and 5.0 ± 0.4 dB mm^–1^ at laser excitation of 1310 nm and 1550 nm respectively). These values provide evidence that the optical signals are transduced unaltered through the crystal. The result is promising for integration of this prototypical material in optical communication devices and fiber-optic cables operating in the O band and the C band of the spectrum. To assess the broadband response from the crystal, a similar method to the one described above was employed, however, a source that delivers a diffraction-limited light in the entire 450–2400 nm range was used (SuperK COMPACT, NKT photonics). As shown in Fig. 3f, the radiation is passively transduced between 1250 and 1600 nm, a range that corresponds to the O, E, S, and C bands, while all wavelengths below 1200 nm are blocked. These properties favor this material as a commercial wide bandpass optical filter. The O and C bands are particularly important for the telecom technology where currently silica fibers are used due to their low optical losses at these bands.

In summary, we report the first example of an organic crystal that transduces light in the near-infrared region with very low optical loss. The material, the amino acid L-threonine, was found to be mechanically robust, as well as photochemically and thermally stable up to 490 K (Supplementary Fig. 7), with Young’s modulus of about 40 GPa (most organic crystals have moduli 1–25 GPa) and very high hardness for an organic crystal. These properties resemble some extreme mechanical properties that are directly associated to higher order packing^48^ such as some tripeptides where the high stiffness is brought about by a super-helical conformation that mimics collagen^49^. Most importantly, crystals of the material selectively transduce light in the O, E, S, and C bands of the near-IR spectrum while blocking wavelengths below and above that range. These properties, and especially the transmission in the O and C bands, could be particularly relevant to applications in telecommunication technologies. Qualitatively, the simplicity of the chemical structure of L-threonine, which lacks multiple chemical bonds between neighboring atoms or benzene rings and is endowed only with functional groups that are capable of very strong charge-assisted hydrogen bonds, can be taken to contribute to the optical transparency of this material. This conclusion goes along with the application of other organic materials, such as simple polymers, as optical waveguides. The unprecedented mechanical robustness and filtering capacity of L-threonine favor this material as a prototypical organic crystalline waveguide that selectively filters out radiation outside its operational range. These observations are poised to open up avenues of research in the active chemistry and materials science communities, and we hope that our results will guide future research efforts directed at the discovery of organic crystalline waveguide materials that are transducive to near-IR frequencies where most of the current fiber-optic-based communications are conducted. In view of materials that will be favorable for practical applications and as a guiding principle for structure selection, the future design of robust molecular crystals that can be used as (short-length) optical waveguides should focus on materials that incorporate hydrogen bonds for stronger cohesion between the structural elements.

## Methods

### Materials

L-threonine was purchased from Sigma-Aldrich. Commercially available solvents were used as received without further purification. Single crystals of L-threonine were grown from a mixture of water and acetone (9:1) as colorless and elongated prisms of up to 1 cm in length and cross-section of ~5 mm^2^.

### X-ray crystallography

The X-ray diffraction data of L-threonine were collected on a Bruker APEX DUO diffractometer equipped with microfocus Mo*K*_α_ radiation (*λ* = 0.71073 Å) and a Photon II detector. The APEX 3 program^50^ was used for determination of the unit cell parameters and data collection. The Bruker SAINT^51^ software package was used to integrate diffraction frames, and the diffraction data were corrected for absorption effects using the numerical method (SADABS)^52^. The structure determination and refinement, using the OLEX2 interface^53^, were performed by using the full matrix least-squares method based on *F*^2^ against all reflections with SHELXL-2014/7^54^. All hydrogen atoms bonded to carbon atoms were fixed using the HFIX command in SHELX-TL. Hydrogens on non-carbon atoms were located from the difference Fourier map. The final CIF was verified by using PLATON^55^ and did not show any missing symmetry. The geometrical calculations were performed by using PLATON^55^ and PARST^56^. The graphics containing the structures were generated using Mercury 3.7^57^, X-Seed^58^, and POV-Ray^59^. Additional details of the data collection and structural refinement parameters are provided in Supplementary Table 1.

### Nanoindentation

The nanoindentation measurements on an L-threonine crystal were performed with an Agilent G200 nanoindenter equipped with an XP head and using a Berkovich diamond indenter. The indenter tip has a nominal radius of about ~30 nm with the pyramidal faces forming an angle of 65.3° with the vertical axis. The indentation was performed using the fixed load measurement and continuous stiffness method with a strain rate of 0.05 s^−1^, an amplitude of 2 nm, and a frequency of 45 Hz. The stiffness and the geometry of the tip were determined by using a Corning 7980 silica reference sample (Nanomechanics S1495-25) before performing indentation. Indentation on all three faces were performed at increasing penetration depths (500−1000 nm). In order to ensure that the tip was fully engaged, the modulus was measured between respective lowest and highest depths. The value of the Poisson’s ratio was assumed to be 0.30. The mechanical parameters and projected area of the crystal were calculated from the load-displacement curves based on the Oliver–Pharr method^60,61^.

### Microscopy

Atomic force microscopy (AFM) measurements on the (00$\bar 1$) face of L-threonine crystals were carried out using a Bruker Dimension Icon system in PeakForce quantitative nanomechanical mapping mode. AFM probe (ScanAsyst-Air) with a spring constant of ~0.4 N m^−1^ and resonance frequency of ~70 kHz was used for scanning the respective crystal surfaces. The deflection sensitivity and the spring constant of the probe were calibrated before proceeding for the imaging. Feedback parameters were auto-controlled, and a scan rate of 0.16 Hz was used to obtain images of good quality. Image processing (leveling data by mean plane subtraction and line profile extraction) on the raw topographies was performed using Gwyddion software^62^.

### Energy framework analysis

The energy frameworks for L-threonine were constructed by using CrystalExplorer17^43,63^. Intermolecular interaction energies, which were partitioned into electrostatic (*E*_elec_), polarization (*E*_pol_), dispersion (*E*_disp_) and repulsion (*E*_rep_) energy components, were calculated based on the B3LYP/6-31 G(d,p) wave functions that were obtained by using the modified CIF. The obtained interaction energies were further utilized to map the network of energy frameworks across different planes. The concept of energy frameworks allows for a better understanding of the intermolecular interactions as it graphically represents the total interaction energies or its individual components as cylindrical tubes joining the molecules^43^. The radii of these cylinders is directly proportional to the strength of the corresponding intermolecular interactions.

### VASP convergence testing of L-threonine crystal structure

Before computing mechanical properties, convergence testing was carried out to ascertain the optimum DFT parameters for relaxing the forces in the crystal structure of L-threonine. All structural optimizations and stress calculations were performed in VASP^64–66^, which uses the projector-augmented wave (PAW)^67^ method with plane-wave basis sets and PAW pseudo potentials. In all calculations, the Perdew-Burke-Ernzerhof (PBE)^68^ generalized gradient approximation (GGA) was used for the exchange-correlational functional coupled with the D3 dispersion-correction^69^. VASP input files were generated using the cif2cell^70^ program. In all cases, a ${{\Gamma }}$-centered Monkhorst-Pack scheme was used to generate a tight K-point mesh with maximum K-point distance set to 2*π* × 0.032 Å^−1^. Convergence testing of the cutoff energy for the planewave basis set was performed using energy cut-offs ranging from 300−1000 eV in increments of 100 eV. Within each self-consistent field cycle, the convergence threshold was set at 1 × 10^−5^ eV and the geometry was considered converged when all forces were below 0.03 eV Å^−1^. Starting with the experimental crystal structure of L-threonine, atomic positions and lattice parameters were optimized simultaneously.

### Computational prediction of the mechanical properties of L-threonine

The convergence testing indicated that a high energy cutoff for the planewave basis set of 1000 eV was necessary to effectively model the forces in L-threonine. Mechanical properties were computed using ElasTool^71^, which interfaces with VASP. Optimized high efficiency strain-matrix sets (OHESS)^72^ were used to determine the full second-order elastic constants. ElasTool uses the stress-strain method to compute elastic constants. Values of −0.03, 0.0, and 0.03 were used to define the strains acting on the crystal. The relaxed structure of L-threonine at a planewave basis set cut-off of 1000 eV was used to define the crystal under zero strain. The OHESS generated crystals under strain were then relaxed at fixed volume using VASP. The planewave basis set energy cut-off was kept fixed at 1000 eV and all other VASP control parameters for the convergence were kept the same as defined above. ELATE^73^ was used to visualize the spatial dependence of the Young’s modulus.

### Molecular electrostatic potential of L-threonine

The assumed molecular conformation of L-threonine was the zwitterion extracted from the variable-cell DFT-D geometry optimization of the experimental crystal structure in VASP. A planewave basis set energy cut-off of 1000 eV was used coupled with the same convergence thresholds for the self-consistent field calculations specified above. The molecular electrostatic potential (MEP) of L-threonine was calculated using GAUSSIAN09^74^ by performing a single-point energy calculation at the B97D/6-31G(d,p) level of theory. The resulting MEP was visualized in GaussView 5.0. Local minima and maxima on the MEP surface (0.0004 au isodensity surface) were calculated using a positive point charge in vacuum as a probe. The calculations lead to the interaction energy (in kJ mol^−1^) between the positive point probe and the surface of the molecule at the point of contact.

### Optical characterization

For the broadband source, a SuperK COMPACT was used. It is a cost-effective supercontinuum white light source delivering diffraction limited light in the entire 450−2400 nm region with a brightness orders of magnitude larger than that of incandescent lamps and with far greater bandwidth than ASE sources or SLEDs. The light is delivered in a single mode fiber terminated with a high quality collimator. For the tunable laser source, the Keysight 8164B Lightwave Measurement System is the ideal mainframe for test solutions of fast and accurate optical applications because it hosts one of Keysight high end tunable laser sources and up to 4 compact modules. In case of the multi-channel lasers, a 4-channel fiber-coupled laser source (MCLS1 Thorlabs) set at maximum laser drive current (100 mA) at 660, 1064 nm was used. For the optical spectrum analyzer (OSA), the light was collected on the opposite crystal terminus with a single mode FC/PC fiber optic patch cable connected to an optical spectrum analyzer (Yokogawa AQ6370D) working in the range 600−1700 nm.

## Supplementary information

Supplementary Information

## Data Availability

The X-ray crystallographic coordinates for the structure reported in this study have been deposited at the Cambridge Crystallographic Data Centre (CCDC), under deposition number 2024959. These data can be obtained free of charge from The Cambridge Crystallographic Data Centre via www.ccdc.cam.ac.uk/data_request/cif. All other data are available from the authors upon request.
