# Supplementary material for: Mechanically robust amino acid crystals as fiber-optic transducers and wide bandpass filters for optical communication in the near-infrared
Source: Nat Commun. 2021 Feb 26;12:1326. doi: 10.1038/s41467-021-21324-y (PMC7910442; doi:10.1038/s41467-021-21324-y)
Supplement: Supplementary file 1 — Supplementary Information [file 41467_2021_21324_MOESM1_ESM.pdf]

Supplementary Information

Mechanically robust amino acid crystals as fiber-optic transducers and wide bandpass filters for optical communication in the near-infrared

Karothu et al.

Supplementary Figures

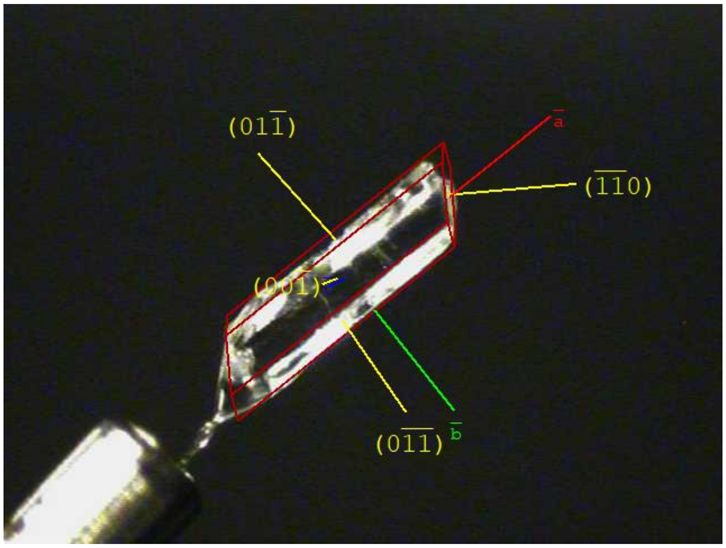

Supplementary Figure 1. Typical habit and face indices of a single crystal of L-threonine.

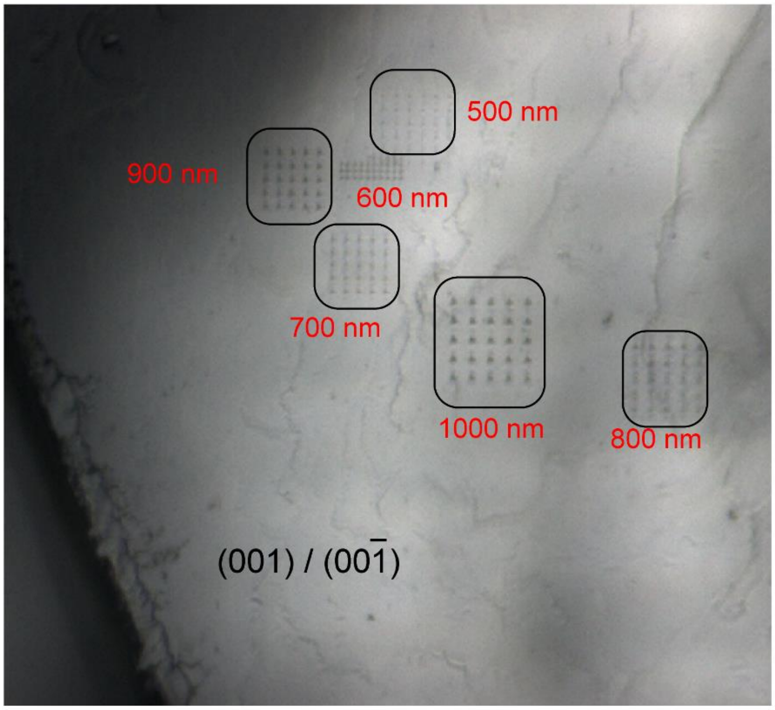

Supplementary Figure 2. Optical image of the surface of L-threonine crystal where nanoindentation was performed at penetration depths from 500 to 1000 nm.

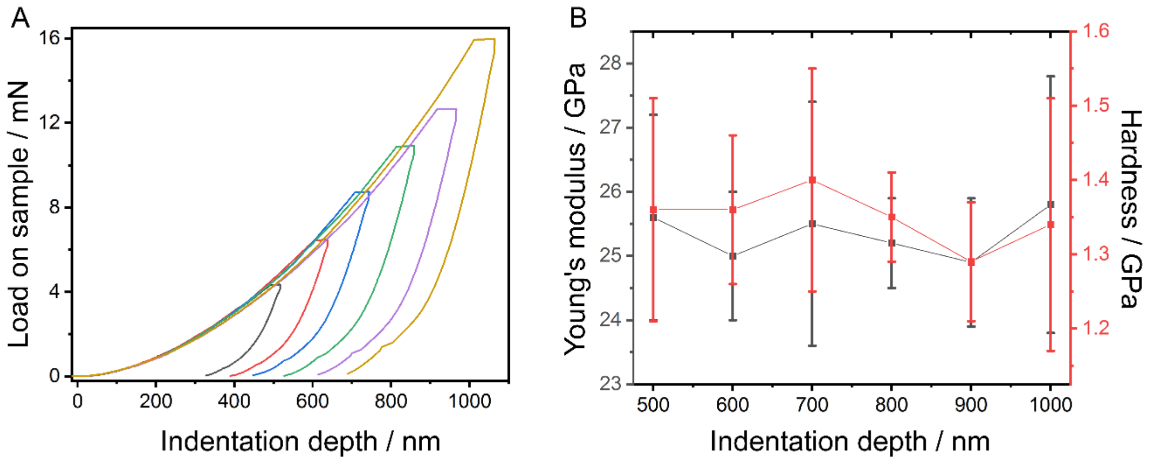

Supplementary Figure 3. Mechanical properties of L-threonine crystals at ambient conditions obtained by nanoindentation. (A) Load–depth curves recorded on the $(\bar{1}\bar{1}0)$ face at different penetration depths. (B) Young's modulus (*E*) and hardness (*H*) based on the curves shown in panel A. *E* = 25.33 ± 1.37 GPa and *H* = 1.35 ± 0.12 GPa. The error bars show the standard deviation of each point.

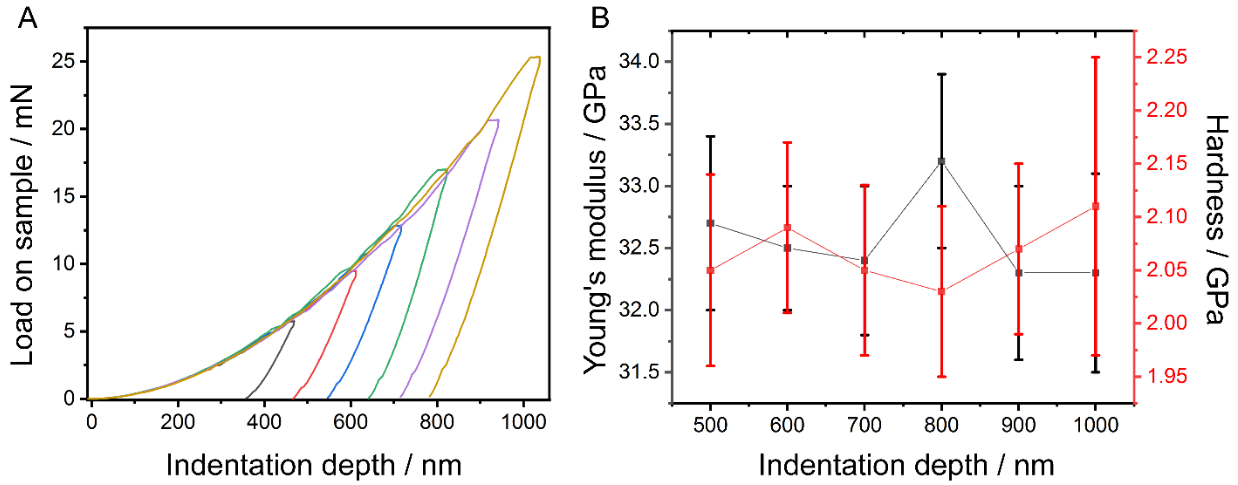

Supplementary Figure 4. Mechanical properties of L-threonine crystals at ambient conditions obtained by nanoindentation. (A) Load–depth curves recorded on the $(0\bar{1}\bar{1})$ face at different penetration depths. (B) Young's modulus (*E*) and hardness (*H*) based on the curves shown in panel A. The determined values are *E* = 32.57 ± 0.67 GPa and *H* = 2.06 ± 0.09 GPa. The error bars show the standard deviation of each point.

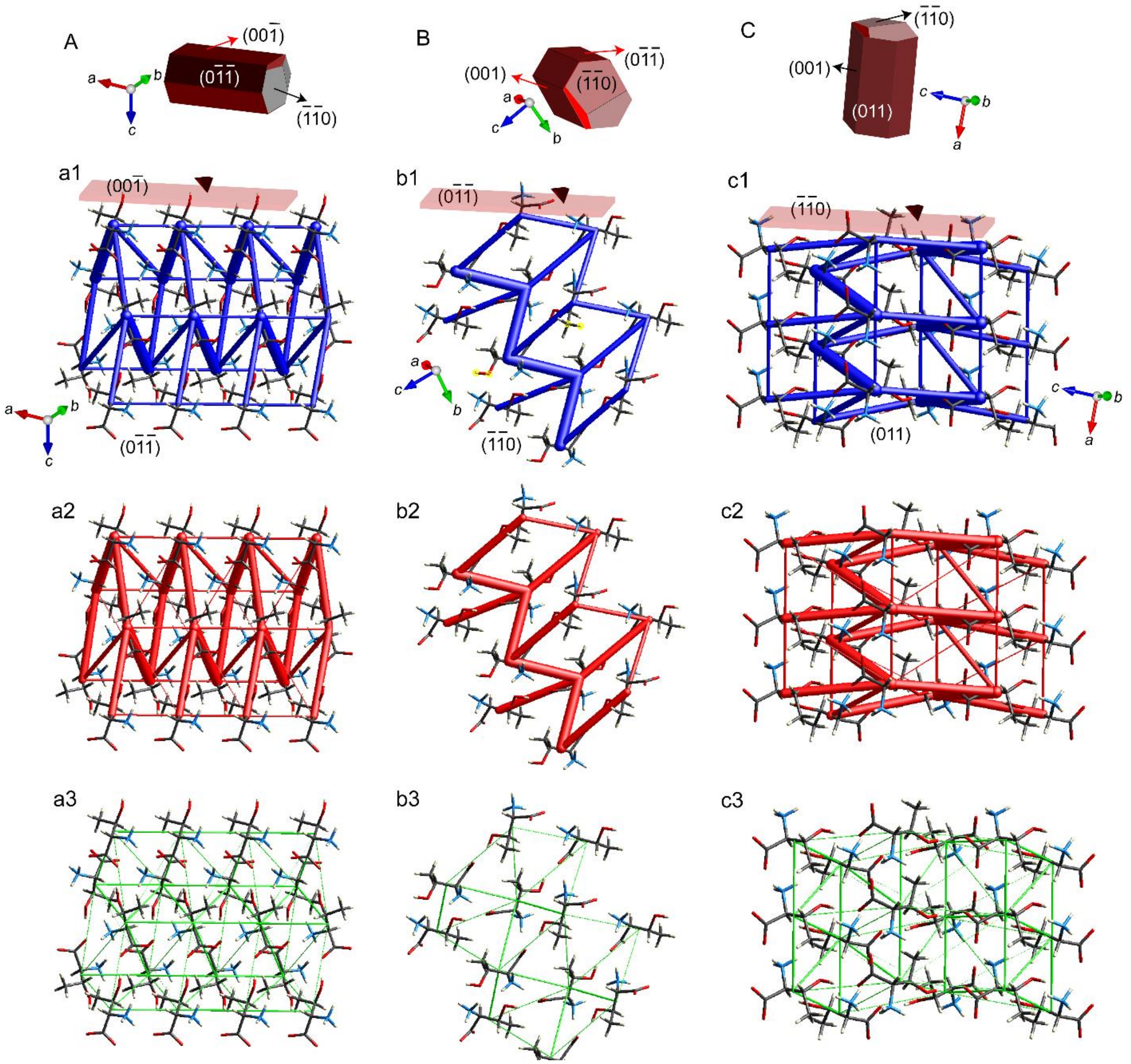

Supplementary Figure 5. Energy frameworks of total energy (blue) along with decomposed electrostatic (red) and dispersive (green) components for L-threonine crystal viewed along different crystallographic directions. The thickness of each tube is proportional to the strength of the respective interaction. The scaling of the tubes is identical in all images where the energy frameworks are shown.

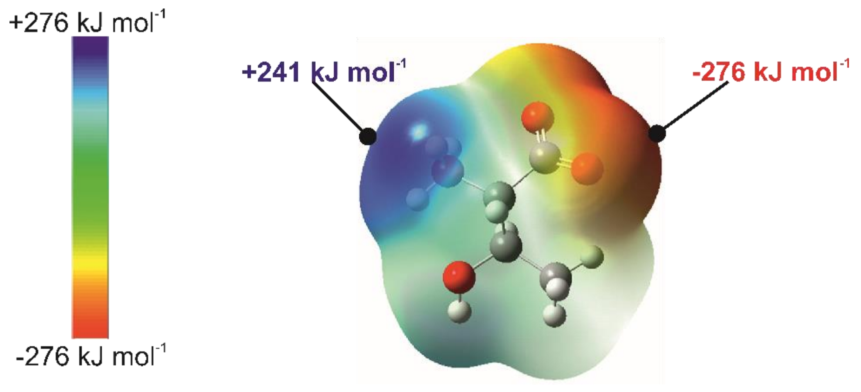

Supplementary Figure 6. Molecular electrostatic potential (MEP) of the L-threonine zwitterion illustrating the highly localized potentials on its surface. The strong charge-assisted hydrogen bonding interactions in the crystal are a consequence of these large potentials on the zwitterion surface.

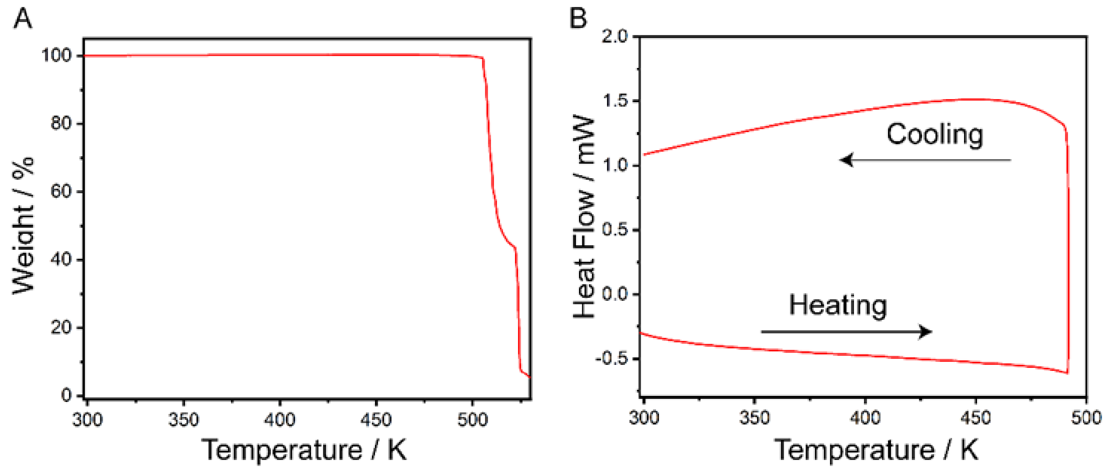

Supplementary Figure 7. Thermogravimetric analysis (A) and differential scanning calorimetry (DSC) (B) of L-threonine crystals showing that the material does not undergo decomposition or a phase transition at temperatures of up to 490 K.

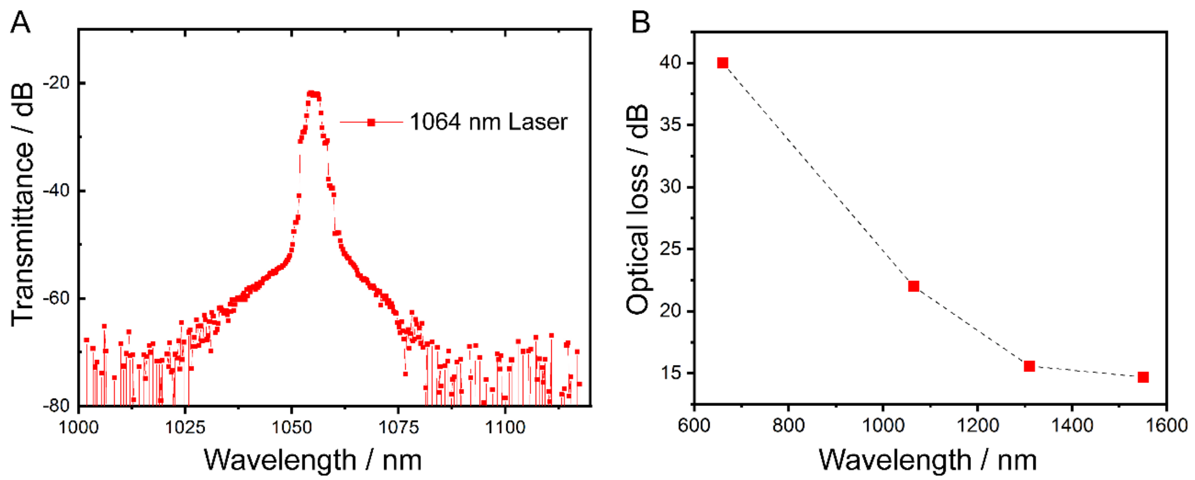

Supplementary Figure 8. Optical waveguiding properties of L-threonine crystals. (A) Optical spectrum of the output at 1064 nm laser excitation. (B) Dependence of the optical losses on the wavelength.

Supplementary Tables

Supplementary Table 1. Crystallographic data and other refinement parameters for the structure of L-threonine

Property	Value
Crystal habit	Elongated prisms
Temperature / K	280
Radiation Source	Mo
Formula weight	119.12
Crystal system	Orthorhombic
Space group	$P2_12_12_1$
a / Å	5.1399(2)
b / Å	7.7335(4)
c / Å	13.6157(6)
α / °	90
β / °	90
γ / °	90
Volume / Å^3	541.22(4)
Z	4
Density / (g cm^{-3})	1.462
μ / mm^{-1}	0.125
F_{000}	256
$h_{min,}$ h_{max}	-6, 6
$k_{min,}$ k_{max}	-9, 9
$l_{min,}$ l_{max}	-16, 16
No. of measured reflections	6880
No. of unique reflections	1028
No. of reflections used	1023
R_{all}, R_{obs}	0.0237, 0.0235
$wR_{2,all}$, $wR_{2,obs}$	0.0603, 0.0601
$\Delta\rho_{min,max}$ / (e Å^{-3})	- 0.106, 0.025
GooF	1.145
CCDC No.	2024959

Supplementary Table 2. Comparison of the experimental and calculated mechanical properties of L-threonine

	**Experimental**	**Calculated (PBE-GGA)**
a (Å)	5.14	5.08
b (Å)	7.73	7.76
c (Å)	13.62	13.57
V (Å^3)	541.22	535.67
Bulk modulus (GPa)		26.80
Young's modulus (GPa)	40.95 ± 1.03	33.11
Shear modulus (GPa)		12.79
Poisson's ratio		0.29
C_{11} (GPa)	-	43.10
C_{12} (GPa)	-	12.50
C_{13} (GPa)	-	13.77
C_{22} (GPa)	-	28.49
C_{23} (GPa)	-	21.40
C_{33} (GPa)	-	74.27
C_{44} (GPa)	-	14.66
C_{55} (GPa)	-	6.97
C_{66} (GPa)	-	9.61
